# A Case of Extra-Uterine Pregnancy

**Published:** 1894-08

**Authors:** J. G. Earnest

**Affiliations:** Atlanta, Ga., Gynecologist Grady Hospital


### A CASE OF EXTRA-UTERINE PREGNANCY.

BY J. G. EARNEST, M.D., ATLANTA, GA.,
Gynecologist Grady Hospital.

On the second day of February, 1894, a lady, age thirty-two, the mother of three children, the last confinement seven years ago, called at my office and stated that she had generally been regular in her menstrual period, but that the period that should have been on the 30th of December, 1893, had not made its appearance until the 10th of January, when she had a slight flow for five or six hours. There was some nausea. The uterus somewhat enlarged, with soft, spongy neck, and the well-marked dusky tint characteristic of pregnancy. I gave it as my opinion that she was pregnant. There was a lump adjacent to the left cornu that seemed to be in the fallopian tube, and suggested the possibility of tubal pregnancy. On this account I asked her to see me in a month again. On the night of 23d of February she was awakened by severe pain in left ovarian region. To use her own expression, she was "paralyzed with pain," and asserts that she could not possibly have given the alarm but for the fact that some one was sleeping with her. Hemorrhage from the uterus accompanied the pains, and quite a number of large clots were passed. Dr. Fisher, of Bolton, was called in and gave a hypodermic injection of morphine that quieted the pain. February 28th I was sent for, and found her comfortable, but quite tender over pelvic region. Her temperature was 100°. The uterus had increased in size, and the mass in left tube seemed now to be nearly as large as an average fist. There was no longer any doubt in my mind as to the nature of the case. I so stated to the patient, and told her an immediate operation would be necessary. However, on account of the absence of her husband the operation was postponed until March 6th, when, at 11 o'clock, assisted by Drs. W. C. Warren and J. McF. Gaston, Jr., of this city, and Dr. Fisher, of Bolton, I opened the abdomen and found the dilated tube dragged down behind the uterus. The adhesions were easily detached and the mass gently lifted out of the pelvis and tied off with a heavy silk ligature. There was no rupture. The abdomen was washed out and closed without drainage. The mass removed was ovoid in shape, very thin walled, about five inches long and two and a half inches in its greatest transverse diameter. It contained a perfect placenta and well-formed fetus of about twelve weeks. It was observed that the uterus was nearly spherical in shape, of a dusky hue, and about two and a half inches in diameter transversely. Two days after operation a free flow of blood from uterus appeared. On the fourth night expulsive pains set in and threw off a mass from the uterus exactly resembling in appearance the placenta found in tube. It was about two and a quarter inches in diameter, about a quarter of an inch thick at the peripheral margin and half an inch thick at the center. One side was smooth and the other presented the rough, serrated appearance that we usually see in a placenta. Hanging to the margin was the torn, ragged edge of a thin membrane. I could make nothing else of it but a placenta, although the mother insisted that she had passed no fetus, but admitted that she had passed numerous large clots, some of which were carried out without examination. This was previous to the operation. It was my intention to bring the specimen home and submit it to a critical and conclusive examination, but in my hurry to catch the train it was forgotten. This I regret, as it places me in a position that I cannot speak with absolute certainty as to the existence of a double pregnancy, but it seems to me that there is little room to doubt that such was the case. Recovery was uninterrupted.

